# StartLink and StartLink+: Prediction of Gene Starts in Prokaryotic Genomes

**DOI:** 10.3389/fbinf.2021.704157

**Published:** 2021-12-09

**Authors:** Karl Gemayel, Alexandre Lomsadze, Mark Borodovsky

**Affiliations:** ^1^ School of Computational Science and Engineering, Georgia Tech, Atlanta, GA, United States; ^2^ Wallace H Coulter Department of Biomedical Engineering, Georgia Tech and Emory University, Atlanta, GA, United States; ^3^ Moscow Institute of Physics and Technology, Dolgoprudny, Moscow, Russia

**Keywords:** gene prediction, inference of translation initiation start, multiple sequence alignment, Kimura distance, integration of omics features

## Abstract

State-of-the-art algorithms of *ab initio* gene prediction for prokaryotic genomes were shown to be sufficiently accurate. A pair of algorithms would agree on predictions of gene 3′ends. Nonetheless, predictions of gene starts would not match for 15–25% of genes in a genome. This discrepancy is a serious issue that is difficult to be resolved due to the absence of sufficiently large sets of genes with experimentally verified starts. We have introduced StartLink that infers gene starts from conservation patterns revealed by multiple alignments of homologous nucleotide sequences. We also have introduced StartLink+ combining both *ab initio* and alignment-based methods. The ability of StartLink to predict the start of a given gene is restricted by the availability of homologs in a database. We observed that StartLink made predictions for 85% of genes per genome on average. The StartLink+ accuracy was shown to be 98–99% on the sets of genes with experimentally verified starts. In comparison with database annotations, we observed that the annotated gene starts deviated from the StartLink+ predictions for ∼5% of genes in AT-rich genomes and for 10–15% of genes in GC-rich genomes on average. The use of StartLink+ has a potential to significantly improve gene start annotation in genomic databases.

## 1 Introduction

Accurate gene finding creates a solid foundation for downstream inference such as the construction of the species proteome, functional annotation of proteins, and inference of cellular networks. Besides providing a start of protein translation, it designates the edge of a gene upstream region populated with signals regulating gene expression (Stormo et al., 1982; de Boer and Hui, 1990; Resch et al., 1996; Laursen et al., 2005).

Gene starts could be experimentally determined by several methods, such as N-terminal protein sequencing (Sazuka et al., 1999; Rudd, 2000; Yamazaki et al., 2006; Aivaliotis et al., 2007; Lew et al., 2011; Zhou and Rudd 2013; de Groot et al., 2014), mass spectroscopy (Rison et al., 2007), and frame-shift mutagenesis (Smollett et al., 2009). Application of these methods is time-consuming; hence, the number of genes with experimentally verified starts is limited. Previous benchmarking studies of gene-finding algorithms used only 2,443 start-validated genes (Hyatt et al., 2010) or 2,925 genes (Lomsadze et al., 2018) known in up to 10 different species.

**FIGURE 1:**
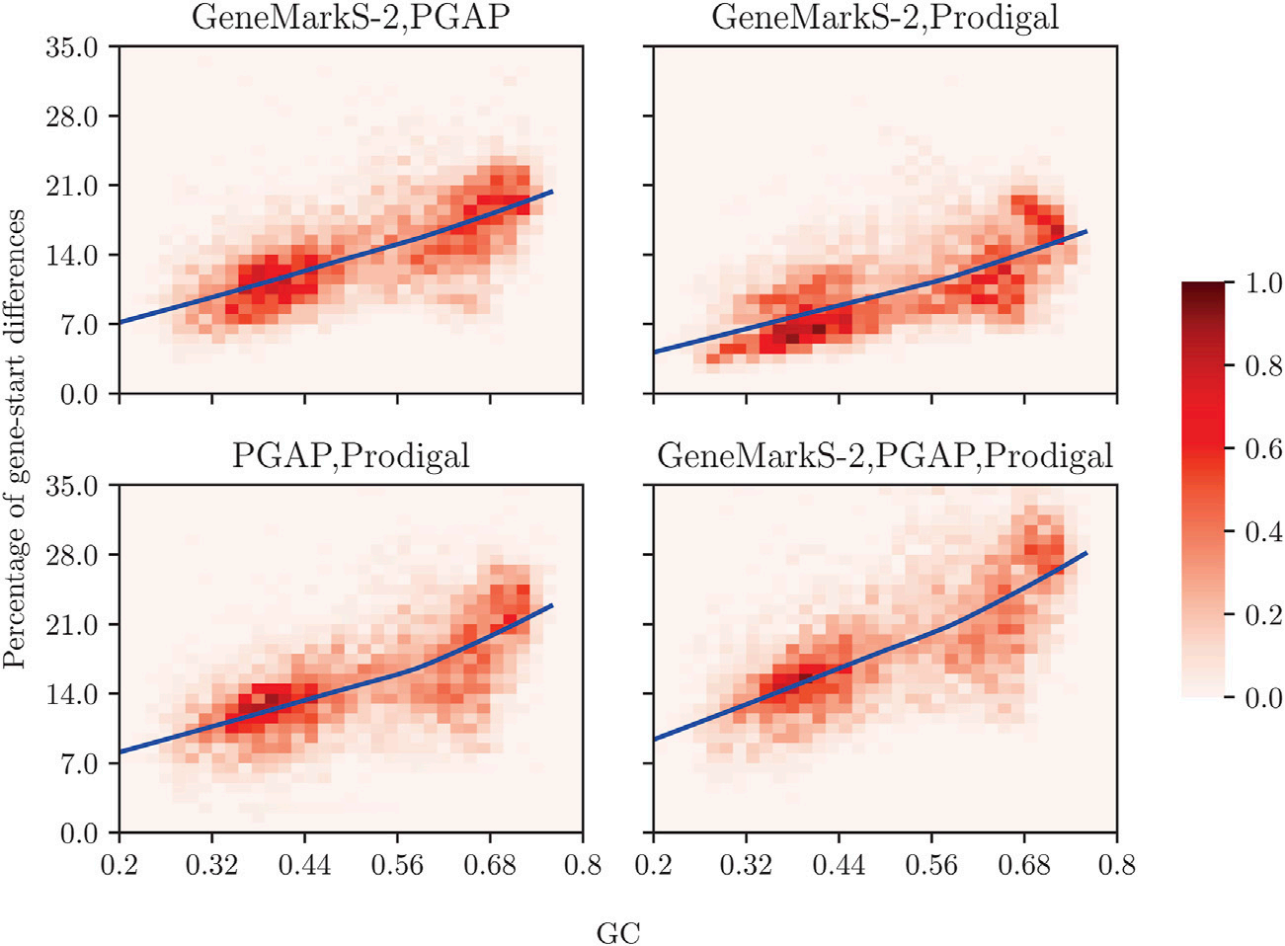
Prodigal, GeneMarkS-2, and NCBI’s PGAP may disagree in gene start predictions. For the NCBI collection of 5,488 representative genomes split between GC-content “bins,” we show the percentage of genes (per genome) with mismatching start such that at least one of the tools has a difference in gene start prediction with the other(s) tool(s). The color of a cell in a graph indicates the number of genomes within the cell as a percentage of 5,488 genomes; the color-coding key ranging from 0 to 1% is given by the bar on the right. The average percentage of genes (per genome) for which gene start predictions differ between the computational tools is shown by solid lines as functions of GC content.

In a computational experiment with 5,488 representative prokaryotic genomes (Figure 1), we have compared gene start predictions made by GeneMarkS-2 (Lomsadze et al., 2018), by Prodigal (Hyatt et al., 2010), and by the PGAP pipeline (Tatusova et al., 2016) guided by alignments of annotated starts of homologous genes. We observed that gene start predictions may differ from annotations on average for 7–22% of the genes in each genome, with high GC genomes showing the larger difference.

Accurate *ab initio* gene start predictions are difficult to be made due to the variability of sequence patterns in the gene upstream regions. While the Shine-Dalgarno pattern (Shine and Dalgarno 1974; Barrick et al., 1994) is dominant in ribosome binding sites (RBSs) of many prokaryotic genomes, other patterns are frequently present, such as non-canonical RBSs. GeneMarkS (Besemer et al., 2001) and Prodigal (Hyatt et al., 2010) were designed to infer RBS models with non-canonical patterns. However, Prodigal’s parameters of the RBS model were optimized for *Escherichia coli* genes with verified starts (Rudd, 2000); this tool is primarily oriented on searching for the canonical Shine-Dalgarno (SD) RBSs. The RBSs located in 5′untranslated regions (5′ UTRs or leaders) are absent in Archaea species leaderless mRNAs, first discovered in *Pyrobaculum aerophilum* (Slupska et al., 2001). Since some antibiotics inhibit translation initiation in leadered transcripts and not in leaderless ones (Brandi et al., 2006; Schuwirth et al., 2006; Kaberdina et al., 2009; Muller et al., 2016; Lange et al., 2017; Sawyer et al., 2018), knowledge of genes with leaderless transcription is instrumental for predicting drug effects on pathogens. To improve gene start prediction in the genomes with leaderless transcription, sequence patterns of promoter sites could be useful. A majority of gene finders have not considered the case when the leaderless and leader-generating transcription could be present in the same genome.

Recently developed self-trained GeneMarkS-2 used multiple models of sequence patterns in gene upstream regions within the same genome. We have found that in 16.4% of archaeal and 61.5% of bacterial genomes (in the NCBI set of 5,007 representative prokaryotic genomes with 238 Archaea and 4,769 bacteria), translation initiation mechanisms have used SD RBSs (Lomsadze et al., 2018). The remaining 83.6% of archaeal species were predicted to frequently use leaderless transcription (along with SD RBSs for some genes). Computational predictions of gene starts in archaeal genomes were supported by experimental observations, for example, for *Halobacterium salinarum*, *Haloferax volcanii*, and *Thermococcus onnurineus* (Koide et al., 2009; Babski et al., 2016; Cho et al., 2017). On the other hand, out of the remaining 38.5% of bacterial species, 10.4% were found to use a non-canonical (non-SD)-type RBSs (e.g., *Bacteroides* (Wegmann et al., 2013)), 21.6% of bacterial species were predicted to use leaderless transcription in up to 40% of transcripts in a genome, for example, *Mycobacterium tuberculosis* (Cortes et al., 2013; Shell et al., 2015; Gualerzi and Pon, 2015; Nakagawa et al., 2017), and in the remaining 6.5% of the bacterial species, the SD-RBS was observed in a small fraction of genes, while the majority of genes had an upstream signal with a very weak sequence pattern that indicated an unknown mechanism of translation initiation, for example, *Cyanobacteria* (Mutsuda and Sugiura, 2006).

A major part of this work was to develop a gene start prediction algorithm, called StartLink, based on multiple sequence alignment. We have not used existing gene-start annotations as well as information on sequence patterns of RBSs or promoter sites (Wall et al., 2011). We have used multiple alignments of *unannotated* syntenic genomic sequences containing predicted coding regions extended to the longest open-reading frames (LORFs). By design, StartLink is a stand-alone predictor of gene starts for all the genes that have a sufficient number of homologs. It is applicable for finding starts of genes residing in short contigs (e.g., assembled from metagenomic reads) for which GeneMarkS-2 (and other whole-genome *ab initio* gene finders) may not perform well due to insufficient volume of sequence data that could be used for supervised or unsupervised training.

On the sets of genes with experimentally verified starts, we have shown that when StartLink and GeneMarkS-2 gene start predictions match each other, a chance of predicting the wrong start is about 0.01. Therefore, we introduced StartLink+, a tool in which output is defined for genes where independent StartLink and GeneMarkS-2 predictions are the same. Genes that have only *ab initio* predictions are missed in the StartLink+ set. We observed that StartLink+ delivered gene start predictions for 73% of genes per genome on average. Comparisons with the gene annotations in databases showed differences of the StartLink+ predictions and annotation in up to 15% of genes in a genome. We argue that the starts of such genes should be reconsidered and, possibly, re-annotated.

## 2 Materials

The five species, bacteria *E. coli* (Rudd, 2000; Zhou and Rudd, 2013), *M. tuberculosis* (Lew et al., 2011), and *R. denitrificans* (Bland et al., 2014), as well as archaea *H. salinarum* and *N. pharaonis* (Aivaliotis et al., 2007), listed in Table 1 had, as of December 2019, the largest numbers of genes with starts verified by N-terminal sequencing (Table 1). These sets of genes were used for the prediction accuracy tests.

**TABLE 1:** Reference clades for the five query species and the sizes of the verified gene test sets (total of 2,841genes).

Species	Clade	# of genomes in the clade	# of verified genes in each species
*Escherichia coli*	*Enterobacterales*	6,311	769
*Halobacterium salinarum*	*Archaea*	1,125	530
*Natronomonas pharaonis*	*Archaea*	1,125	282
*Mycobacterium tuberculosis*	*Actinobacteria*	8,097	701
*Roseobacter denitrificans*	*Alphaproteobacteria*	4,720	526

As of November 4, 2019, NCBI’s RefSeq database had over 183,689 annotated prokaryotic genomes. To reduce the time for search for homologs, the search space could be limited to a clade the query species belongs to (Table 1). Among genomes with the same taxonomy ID, we selected the one with the most recent annotation date. In the selected genomes, all longest open-reading frames (LORFs) of annotated genes were extracted and translated, and a BLASTp database was built.

We have conducted computational experiments with genomes from four clades (with the numbers of randomly selected genomes given in parenthesis): *Archaea* (97), *Actinobacteria* (95), *Enterobacterales* (106), and *FCB* group (96). The clade selection was guided by the study of patterns in gene upstream regulatory regions (Lomsadze et al., 2018). Archaeal genomes have large numbers of genes with leaderless transcription. Clade *Actinobacteria* has predominantly high-GC genomes with a significant number of genes with leaderless transcription. The *Enterobacterales* clade has mostly mid-GC genomes that carry genes with an RBS of the Shine-Dalgarno type. Finally, the *FCB* group has low-to-mid-GC genomes that carry genes with a “non-canonical” AT-rich RBSs (Lomsadze et al., 2018).

The prokaryotic genome collection of the NCBI includes a description of 5,488 genomes representative of the whole database. We used this set to show the extent of differences in prokaryotic gene start predictions made by the state-of-the-art tools.

## 3 Methods

### 3.1 Metrics for Gene Start Prediction Performance

Given a test set of genes, set G , we consider its subset S for which a particular algorithm predicts gene starts. We define the following measures: *accuracy*, Acc(S, G); *error rate*, Err(S, G); and *coverage*, Covr(S,G):

$$\begin{array}{l} Acc\left(\text{S},\text{G}\right)=100*\frac{M_{5}\left(\text{S},\text{G}\right)}{M_{3}\left(\text{S},\text{G}\right)},\\ Err\left(\text{S},\text{G}\right)=100-Acc\left(\text{S},\text{G}\right),\\ Covr\left(\text{S},\text{G}\right)=100*\frac{M_{3}\left(\text{S},\text{G}\right)}{\left|G\right|}. \end{array}$$ (1)

Here, $M_{5}\left(\text{S},\text{G}\right)$ and $M_{3}\left(\text{S},\text{G}\right)$ are the numbers of genes in S that match genes in G by both 5′ and 3′ ends, and only by 3′ ends, respectively. With respect to commonly used measures of sensitivity and specificity (e.g., Lomsadze et al., 2018), we have to note that Acc(S, G) is measured for predictions of gene starts. Correct prediction of prokaryotic gene starts is a significantly more difficult problem than the prediction of gene-reading frames and hence the positions of 3′ end. Therefore, in comparison of two advanced gene-finding tools 1 and 2, we could assume that sets *M*_*3*_ *(S*_*1*_ *,G)* and *M*_*3*_ *(S*_*2*_ *,G)* are the same. Next, we observe that the number of predictions made by a given tool *i* is equal to the number of genes in set *M*_*3*_ *(S*_*i*_ *,G).* Therefore, *Sn*_*i*_ = 100*M_*5*_ *(S*_*i*_ *,G)/M*_*3*_ *(S*_*i*_ *,G) = Sp*_*i*_. Notably, an error in gene 5’ end prediction makes both false negative and false positive at the same time. Thus, in our case, the values of *Sn* and *Sp* are numerically equal to each other and to those of Acc(S, G).

### 3.2 StartLink

**FIGURE 2:**
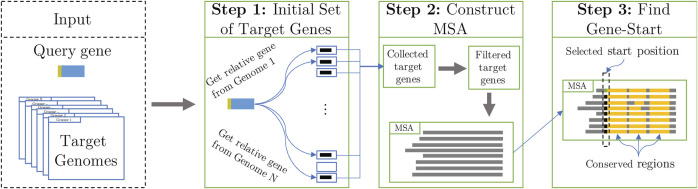
High-level schematic of the StartLink workflow (Figure 5 shows details of step 3).

The task is to identify the start codon of a prokaryotic gene within its longest open-reading frame (LORF) embedded in a nucleotide sequence Q (query). The StartLink algorithm identifies and uses syntenic genomic sequences upon making the following three steps (Figure 2):

Select a set of target genomic sequences defined by the search for query Q.
Eliminate evolutionarily too close and too remote to Q target sequences as well as the target sequences too close to each other and construct a multiple sequence alignment (MSA).
Select gene start among possible candidates within the LORF in query Q.

#### 3.2.1 Step 1: Finding Homologs

A protein product of a gene predicted by GeneMarkS-2 in the query sequence is used in the Diamond BLASTp (Buchfink et al., 2015) to find a set of target proteins and genes (described in more detail below) that have significant similarity to the query. We remove any target whose pairwise protein alignment with the query does not cover more than 80% of either the query or target sequences. This step helps eliminate targets whose alignments with query do not cover the areas close to the target gene start.

#### 3.2.2 Step 2: Selection of Target Proteins and Construction of a Multiple Sequence Alignment of Syntenic Nucleotide Sequences

With the set of target proteins and their genes in place, we proceed to build an informative MSA for gene-start inference. Each target gene is extended to LORF and translated into the amino acid sequence. The protein MSA is constructed by the Clustal Omega algorithm (Sievers and Higgins, 2018) from 50 randomly selected translated LORFs along with the translated LORF of the query. Next, the algorithm constructs pairwise alignments of the LORF nucleotide sequences guided by protein MSA within the gene regions. Based on the pairwise alignments, the algorithm computes the query-to-target and target-to-target evolutionary distances by using the Kimura 2-parameter model (Kimura, 1980):

$$d_{\mathrm{AB}}=-\frac{1}{2}ln\left[\left(1-2P-Q\right)\sqrt{1-2Q}\right],$$ (2)

where P and Q are the fractions of positions in the alignment with transition or transversion mutations, respectively. The Kimura distance is usually computed for a global alignment of two DNA sequences. We have observed that for closely related genomic sequences, a local alignment (derived from the readily available BLASTp output) could provide sufficiently accurate distance value, thus saving the effort of realigning sequence pairs (see Supplementary Note 6).

Aligned syntenic genomic sequences should carry conserved patterns downstream from true gene starts. However, the presence of distant from query sequences ( $d_{AB\;}>0.5)$ leads to the insertion of many gaps in MSA downstream from the gene starts, thus disrupting the pattern of conservation (see Supplementary Materials). On the other hand, if two syntenic sequences are too similar ( $d_{AB}<0.1)$ , then one of them is redundant and could be removed. Therefore, we select target sequences that fall inside the $d_{AB\;}$ range [0.1, 0.5] with respect to the distance to query.

**FIGURE 3:**
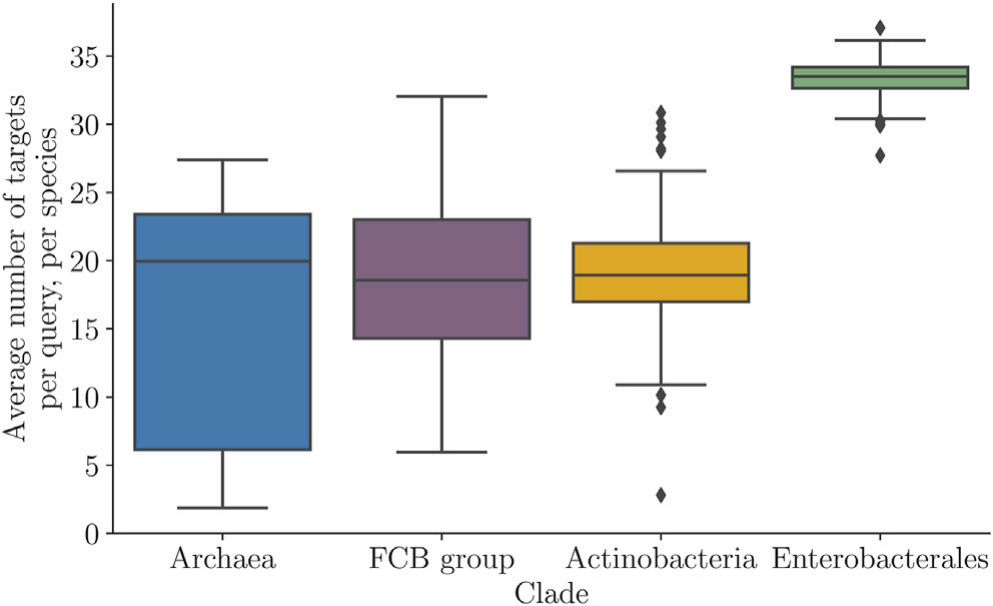
Average number of targets per query in the StartLink runs. The average was computed per genome and shown for each of the four clades (in the whole set of 443 query genomes).

The number of target sequences in the final MSA varies from 10 to 50, and the average number of MSA sequences is clade-specific (Figure 3). We observed that MSAs with low numbers of targets (e.g., about 10) still contain informative sequences.

Note that selection of reference target genes in StartLink is gene-specific and takes into account that different genes evolve with different speeds. This approach attempts to produce similar distributions of Kimura distances despite variations in the gene-specific and clade-specific speeds of evolution.

#### 3.2.3 Step 3: Identification of the Gene Start in a Query Sequence

The algorithm predicts the gene start by analyzing patterns of conservation in the MSA at one of the three following steps.

1) Search for conserved blocks in protein MSA and the simplest case of the gene-start identification.

Given a protein MSA constructed from the translated LORFs of a query and its targets, the algorithm searches for the left-most *block with a high conservation score* (see below). We assume that the nucleotide sequences of the true genes in the corresponding set of nucleotide sequences do not overlap with the upstream genes. If a left-most protein block with a high score is detected and there is only one gene start candidate in the nucleotide query upstream of the block, this candidate is predicted to be the gene start. Otherwise, the algorithm proceeds to (B). Note that the start assignment in (A) does not require conservation of the start candidate itself.

For a protein MSA block of length r (where r=10aa not including possible N-terminal), a conservation measure (identity) score is computed by the formula:

$$S_{blk}\left(i,r\right)=\frac{1}{r\times {\left(N-1\right)}^{2}}\sum_{m\neq n}\sum_{j\in J\left(i,r\right)}H\left(m,n,j\right),$$ (3)

where J(i) is the set of r positions downstream of position i, with no gap in the query; H(m,n,j) is 1 if and only if sequences m and n match each other at position j in the alignment; and N is the total number of sequences in MSA. A block with $S_{blk}\left(i,r\right)$ larger than 0.5 is identified as conserved. This threshold corresponds to the uninformed, majority-vote approach, which is a reasonable option when limited ground-truth data are available.

2) Identification of the gene start in the presence of overlapping genes

**FIGURE 4:**
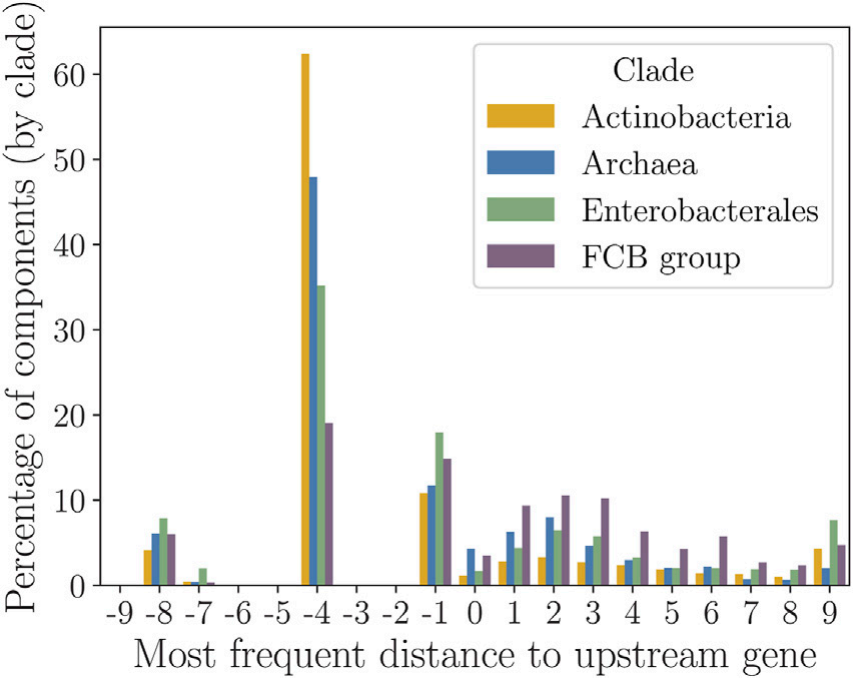
Frequency histogram of the most frequent intergenic distance x between same-strand genes in the MSA-defined components. The x value is in the range from −10 to +10 nt.

If a query LORF overlaps with the 3′ end of the upstream gene (which is easy to be determined), such an overlap is likely to appear in syntenic sequences (at a sufficiently close evolutionary distance, see Figure 4). It was observed that ATG, GTG, or TTG codons of a LORF situated near the 3′ end of the upstream gene have elevated frequency of being true starts (Lukashin and Borodovsky, 1998; Huber et al., 2019). It is plausible that the ribosome can efficiently reassemble at such a gene start upon completing the translation of the upstream gene. StartLink attempts to identify a conserved gene-start candidate in the MSA within 9 *nt* distance near the 3’ end of the upstream gene. The conservation score for the candidate with MSA position *i* is defined by the fraction of targets that have gene-start candidates within 6nt distance from position *i*. Formally, the identity score for position i is defined as

$$S_{5^{\prime }}\left(i,x\right)=\frac{1}{N}\sum_{j=1}^{N}\left(G\left(i,j,x\right)-P\left(i,j\right)\right),$$ (4)

where

$$G\left(i,j,x\right)=I\left\{\left|\left\{ATG,GTG,TTG\right\}\cap^{\text{​}}Neigh\left(i,j,x\right)\right|\geq 1\right\}.$$ (5)

Here, I{⋅} is the indicator function, |⋅| computes the size of a set, and Neigh(i,j,x) is the set of codons within a distance of x codons around position i in sequence j . Thus, G(i,j,x) is 1 if an ATG, GTG, or TTG exists in the neighborhood, and 0 otherwise. The term P(i,j) penalizes for the appearance of the codons being synonymous to GTG, or TTG, but not serving as start codons; P(i,j)=1 if such a codon exists in position i of sequence j in the MSA, and 0 otherwise. $If\;S_{5'}\left(i,x\right)$ > 0.5 the candidate is selected as a predicted start; otherwise, the algorithm moves to (C).

For additional justification of step C, we introduced the following consideration. In the analysis of a large set of query genes and their homologs (targets), we found that if a query gene was overlapped by the upstream gene (in the same strand) or if the upstream intergenic region was very short (less than 10 nt), then such a configuration was preserved for genes in genomes of closely related species. Let us consider a query gene along with its targets defined by similarity search and included in the MSA (N sequences, N > 10); this set is called *a component*. Let D(n) be the length of the intergenic region from the end of the upstream gene to the start of the downstream gene, n, and let x be the most frequent D(n) observed in the component (i.e., the mode). Then, the measure of conservation of the intergenic region being x nucleotides long is defined as

$$DC\left(x,f\right)=\frac{1}{N}\sum_{n=1}^{N}I\;\{x-f\leq D\left(n\right)\leq x+f\},$$ (6)

where I(⋅) is the indicator function and the margin f determines the stringency of conservation. The DC value could be interpreted as a probability that in any sequence that belongs to the component, the upstream gene is located x±f *nt* away, where x is the most frequent distance in the component. The distribution of the measure of the conservation was computed and presented in the Results section (Supplementary Figure S11).

3) Identification of the gene start in a general case

C-1: If multiple gene-start candidates are present in the query LORF upstream to the left-most MSA block of conserved amino acids, the $S_{5'}$ scores of the candidates are computed and screened from the LORF 5’ end downstream. If a candidate has $S_{5'}$ > 0.5, then the algorithm moves to C-2. Otherwise, it moves to the next candidate. If all candidates have been exhausted, then the algorithm quits without selecting any candidate as a predicted gene start.

**FIGURE 5:**
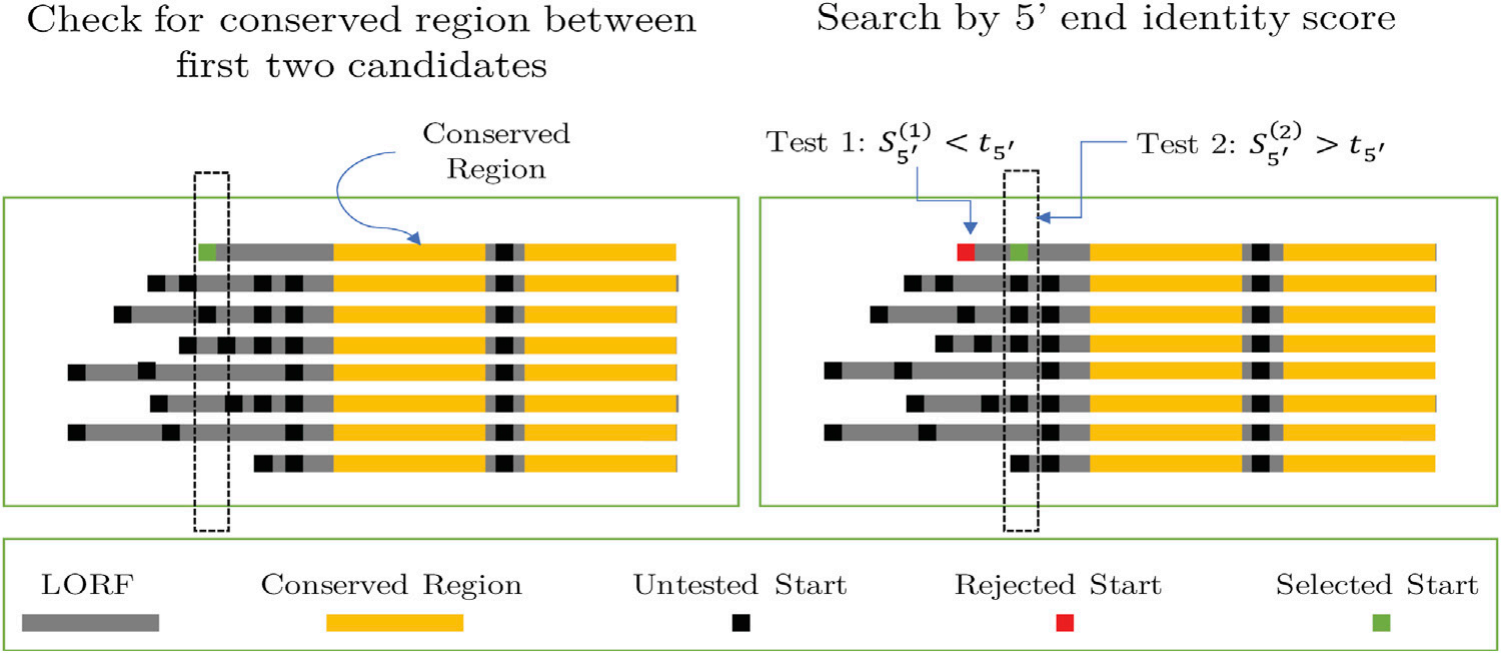
Use of MSA to identify a start of a gene in the query sequence (top sequence in each MSA). Left panel: Step A: the left-most conserved block is detected, with a single gene-start candidate located upstream. Right panel: Step C: Candidate start codons are screened to find those with conservation score $S_{5^{'}}\left(i,x\right)\;$ above the threshold $t_{5'}=0.5$ (Supplementary Note 3).

C-2: To avoid missing a true start downstream of the candidate selected in C-1, the algorithm searches for a candidate with a highest $S_{5'}$ score- $S{'}_{5'}$ in the 30 nt region downstream. If $S{'}_{5'}$ > 0.5 and if there is a conserved block (of any length up to 10 *aa*) between the two candidates, then the upstream candidate is selected; otherwise, the downstream candidate is identified as the start (Figure 5).

### 3.3 StartLink+: A Pipeline Combining StartLink and GeneMarkS-2

StartLink+ runs both GeneMarkS-2 and StartLink. Genes whose starts are predicted in the same position by both tools are selected. This set of starts is reported as the output of StartLink+. Since an error of StartLink+ would occur in an event that two independent tools would make the same erroneous prediction, the expected error rate is proportional to a product of probabilities of an error of each tool.

## 4 Results

### 4.1 Accuracy Assessment on Genes With Experimentally Verified Starts

In the set of genomes containing genes with verified starts, we selected the genes with StartLink+ predictions. The coverage values, that is, percentage of genes in each set for which a particular method generates gene start predictions, are shown in Table 2 for StartLink, GeneMarkS-2, and StartLink+.

**TABLE 2:** Error rates in gene start predictions (%) and gene set coverage determined for StartLink, GeneMarkS-2, and Prodigal as well as for their combinations: StartLink and GeneMarkS-2, that is, StartLink+, StartLink and Prodigal, and GeneMarkS-2 and Prodigal, on sets of genes with experimentally verified starts. The sizes of the sets are shown in Table 1.

	StartLink		GeneMarkS-2		Prodigal	
	Error rate	Coverage	Error rate	Coverage	Error rate	Coverage
*E. coli*	4.45	99.35	3	99.74	2.34	100
*H. salinarum*	2.73	89.81	1.32	100	2.84	99.81
*M. tuberculosis*	6.86	85.31	9.6	99.57	11.05	99.43
*N. pharaonis*	2.11	90.16	0.95	100	1.59	99.68
*R. denitrificans*	4.81	90.87	3.43	99.81	4.94	100
Average	4.19	91.1	3.66	99.82	4.55	99.78

	StartLink+		StartLink and Prodigal		GeneMarkS-2 and Prodigal	
	Error rate	Coverage	Error rate	Coverage	Error rate	Coverage
*E. coli*	0.83	93.63	0.69	94.15	1.08	96.62
*H. salinarum*	0.43	87.17	0.44	85.66	0.39	96.79
*M. tuberculosis*	1.32	75.75	1.35	74.04	3.95	86.73
*N. pharaonis*	0	87.62	0	86.98	0.64	98.73
*R. denitrificans*	0.45	84.6	0.68	83.84	1.01	94.11
Average	0.61	85.75	0.63	84.93	1.41	94.6

The GeneMarkS-2 coverage deviated from 100% in a given set when the gene finder did not predict one or more genes as a whole. StartLink was missing genes where neither A or B or C steps produced start predictions. In addition to genes missed by either GeneMarkS-2 or StartLink, StartLink+ missed genes where gene starts predicted by GeneMarkS-2 and StartLink do not match. The lowest StartLink+ coverages ∼75% were observed for *M. tuberculosis* and *R. denitrificans*.

The error rates of gene start prediction by StartLink, GeneMarkS-2, and StartLink+ were computed by a comparison of the predictions with the coordinates of verified gene starts (Table 2). The error rates of StartLink+ were as low as 0.61% on average. The reduction of the error rates observed for the two independent tools was significant. Particularly, for *M. tuberculosis*, StartLink and GeneMarkS-2 error rates were ∼6.9 and ∼9.6%, respectively. The error rate of StartLink+ was ∼1.3%.

Gene start prediction in high GC genomes is known to be challenging. Three genomes in Table 2 had high GC content: *H. salinarum* (65%), *N. pharaonis* (63%), and *M. tuberculosis* (66%); the StartLink+ error rate was 0.6, 0.0, and 1.32%, respectively.

The percentage of verified genes where predictions of StartLink, StartLink+, GeneMarkS-2, and Prodigal deviate from experimentally confirmed starts is shown in Table 2. We ran StartLink+ on 443 prokaryotic genomes from the four prokaryotic clades (Table 1). The StartLink+ error rate in gene start predictions (albeit with the reduction in coverage) was the lowest (Table 2). Therefore, StartLink+ is expected to generate for a given genome a large set of genes with starts reliably determined. Note that if we select a subset of genes that is common for StartLink, GeneMark-S, and Prodigal in terms of the same 3’ ends, with the elimination of 289 genes out of 2,841, the results shown in the top row of Table 2 will hold, with GeneMarkS-2 having the lowest error rate (data not shown).

For comparison, we also show the percent of errors in gene start prediction made by each of the three tools, StartLink, GeneMarkS-2, and Prodigal, on a subset of experimentally verified genes predicted by all three tools (Supplementary Table S2). The accuracy pattern observed in Table 2 holds.

**FIGURE 6:**
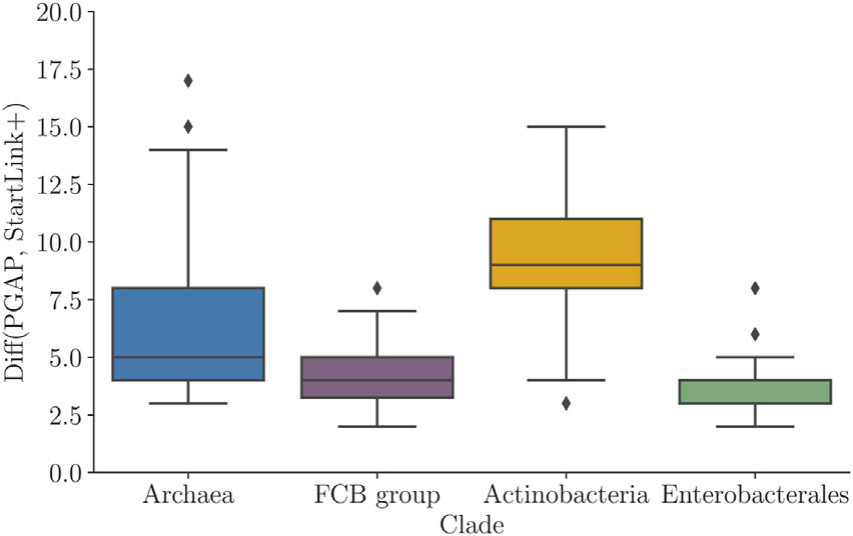
Percentage of genes per genome having differences in PGAP and StartLink+ predictions of gene-start positions. Distributions of average values per genome for prokaryotic clades (Table 1).

We compared the predicted gene starts with the PGAP annotation. We observed that the average percentage of genes with differences in gene-start positions between PGAP and StartLink+ was non-uniformly distributed among the clades (diff values in Figure 6). Particularly, in the *Actinobacteria* genomes, that difference reached up to 15% of genes per genome, with an average of around 10%. On the other hand, the average difference dropped to about 4.5% in genomes of the *FCB group* and to ∼3% in *Enterobacterales* genomes. Notably, there were inter-clade differences in average genome GC contents, for example, *Actinobacteria* (high GC) and *Enterobacterales* (mid GC), as well as in clade-specific abundance of leaderless transcription.

**FIGURE 7:**
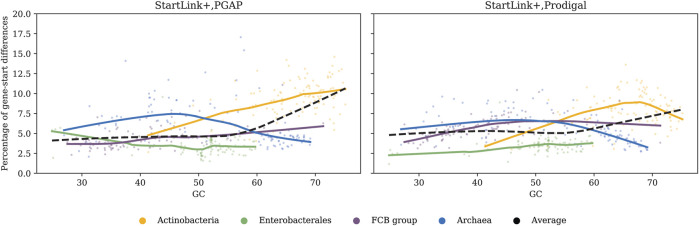
Percentage of genes per genome where predicted start positions differ between PGAP and StartLink+ (left) and Prodigal and StartLink+ (right) as a function of genome GC content. The analysis was done for 443 genomes from the four clades (Table 1).

Of interest is how genome GC content affects the genome-specific percentage of genes with differences in start positions defined either by PGAP and StartLink+ or by Prodigal and StartLink+ (Figure 7). In both cases, the percentage of In *Archaeal* genes with differences was highest in mid-GC genomes and decreased in high-GC and low-GC genomes. In *Actinobacteria*, the percentage of genes per genome increased with GC increase for both Prodigal and PGAP, but beyond 67% GC, it began decreasing for Prodigal. The gene-start annotation in PGAP gives preference to the location of gene starts that correspond to the conserved signature inferred from *annotated* starts of known genes (Tatusova et al., 2016). This method depends on earlier annotations and is prone to transferring errors.

### 4.2 Gene Start Prediction Accuracy at Each StartLink Step

As previously mentioned, StartLink output was generated at one of the three possible steps, namely, A, B, or C, depending on the gene-specific sequence alignment configuration. We used *sets of genes with verified starts* to assess error rates of both StartLink and StartLink+ at every three steps. We also computed the percentage of gene-start differences between StartLink+ prediction and PGAP annotation at each step.

**FIGURE 8:**
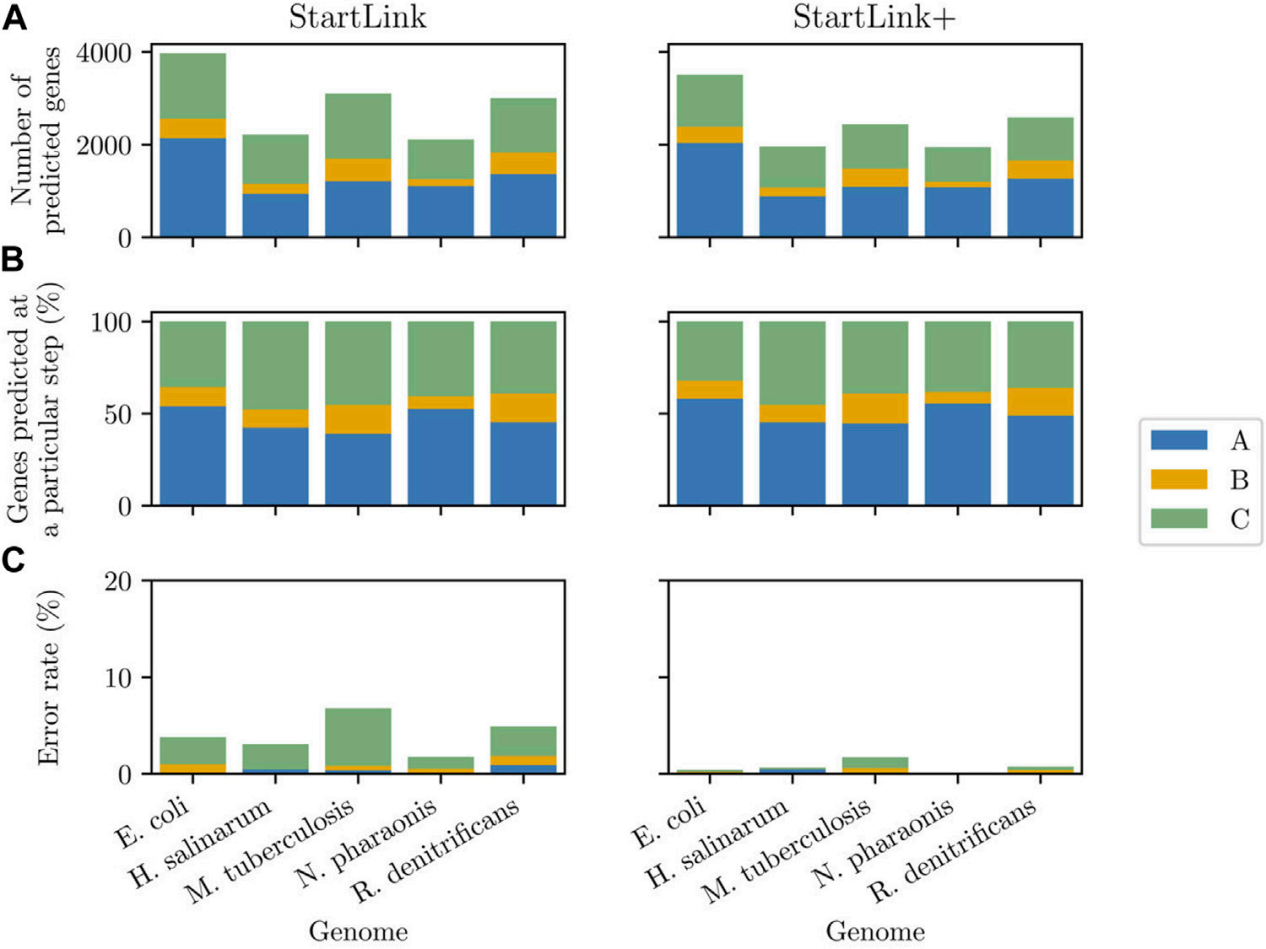
Left panels: The gene start prediction error rate of StartLink observed at each of the steps **(A)**, **(B)**, or **(C)** computed for the sets of genes with verified starts in the five species (top), the percentages of the genes in a given genome predicted in StartLink by step **(A)** alone, by steps **(A)** and **(B)**, and by all steps together (middle), and the absolute numbers of the predicted genes depicted in the middle sub-panel (bottom). Right panels: Same data types as in the left panels computed for StartLink+.

At step A, the observed error rate for five species was consistently low, close to zero (Figure 8, bottom left panel). This result matched the logic of step A where the predictions were made with strong evidence for a particular gene start; ambiguous cases were delegated to the subsequent steps. However, the error rates observed at step B were rather low as well. Among the genes with verified starts, a very few genes had a closely situated or overlapping upstream gene that would require going through step B (Figure 8, top two panels). Particularly, for *N. pharaonis*, step B was a final step for only seven genes.

Gene start prediction in the three steps of StartLink+ had lower error rates, as would be expected (Figure 8, bottom right panel). Interestingly, the observed patterns of differences between StartLink+ predictions and the PGAP annotation were similar but not the same in the four prokaryotic clades (Supplementary Figure S10). The differences were consistently smaller at step A (in the range of 2–6%) than at steps B and C (5–12%). Similar patterns were found when comparing StartLink+ to Prodigal (data not shown).

### 4.3 Conservation of Gene Overlaps in Syntenic Regions

Computations with formula (9) did provide quantitative evidence for the presence of conservation of the length x of gene overlaps and short intergenic regions. The DC value was decreasing when x was increasing (Supplementary Figure S11). Thus, we saw that gene overlaps tend to be conserved within components.

To determine the percentage of components (per clade) that fell for each value of x , we zoomed in into the range of x *nt* between -10 and 10 (Figure 4). Most components within that range had 4 *nt* gene overlap, followed by 1 *nt* overlap. This tendency was particularly pronounced in *Actinobacteria*, where more than 60% of components had 4 *nt* gene overlap. In the *FCB* group, components with 4 *nt* gene overlap constituted only 20% of all components. This decrease with respect to *Actinobacteria* could be related to the presence of AT-rich non-canonical RBSs in the gene upstream regions in genomes of the *FCB* group (Lomsadze et al., 2018). Such AT-rich RBSs could have been evolved and maintained in lower GC non-coding regions rather than inside the upstream protein-coding gene with higher GC. The observed preferences for both -4 and -1 overlaps were in agreement with the previous works suggesting that gene-start positions close to the 3’ ends of the upstream genes were favored in evolution (Lukashin and Borodovsky, 1998; Huber et al., 2019).

### 4.4 Analysis of Distributions of the Kimura Distances

StartLink infers gene-start position from analysis of patterns of conservation in nucleotide sequences of syntenic LORFs. The LORF sequences containing homologous genes are selected for a query by the BLASTp search in the BLAST database precomputed for the given clade. Multiple alignments of LORFs are analyzed to detect changes of the positional frequency of nucleotides (the conservation pattern) upon crossing the position of gene start from the intergenic region to a gene or from a gene in one reading frame to a gene in another frame in the same DNA strand (in a gene overlap). The task of detection of the conservation pattern change point may not be solved satisfactorily if the reference LORFs are evolutionarily too close or too distant from the query LORF. Therefore, we have analyzed the dependence of the accuracy of StartLink on the range of evolutionary distances between query and targets measured by the Kimura model, as well as distances between targets.

The clade-specific accuracy of StartLink could depend on the clade-specific organization of groups of homologous genes or proteins. We analyzed distributions of the Kimura distances between query genes and their targets across different clades (in the distance range [0.1, 0.5]).

Regardless of the nature of the differences in the Kimura distance distributions (caused by the variability of the speed of evolution or inhomogeneity of the database sampling), the similarity-based method, such as StartLink, had to be designed to work in a non-uniform space of homologs (Supplementary Note 2).

A set of orthologs found by similarity search for a given query *q* was filtered prior to MSA construction. This set had minimum and maximum values of the Kimura distances to the query. These two values made a vector (min K(*q*), max K(*q*)), and the frequency distribution of these vectors within the triangular space was depicted by the contour plots (Supplementary Figure S12). The plots show clear differences in the distributions of the “minmax” vectors among queries in each clade. For example, most query genes in *Enterobacterales* had the “minmax” vectors of the Kimura distances close to the extreme one [0.1, 0.5].

**FIGURE 9:**
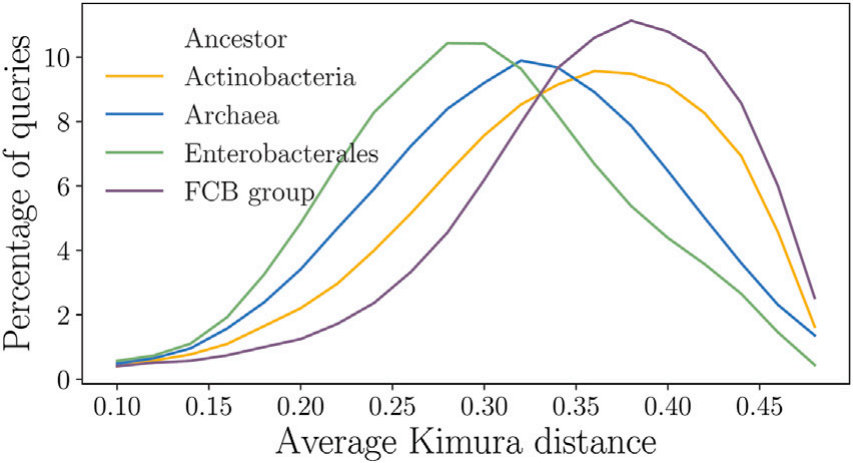
Distributions of the average Kimura distances from a query to the homologs for the four clades. The y-axis shows the percentage of queries having a particular average Kimura distance to their homologs.

In *Actinobacteria* and in the *FCB* group, however, large fractions of the query genes had the closest relatives at a rather long distance with the minimum Kimura distance varying from 0.1 to 0.4. Therefore, the average Kimura distance per query was 0.38 for *Actinobacteria* and the *FCB* group compared to 0.23 for *Enterobacterales* (Figure 9). We observed that the homologs of genes of *Enterobacterales* species span uniformly a broad range of the Kimura distances (from the respective query genes). Such distributions produced a robust performance of StartLink as well as high coverage of genes in a query genome by the StartLink predictions.

For the set of genes with verified starts, we found error rates of StartLink and especially StartLink+ are uniformly low regardless of the Kimura distance range (see Supplementary Figures S2, S3). We also observed that deviations of the StartLink+ predictions from the PGAP annotation were in the same range regardless of variations in min and max values of the range of the Kimura distance between queries and targets (Supplementary Figure S5).

### 4.5 Variability of the BLAST Hit Distributions Across Different Clades

**FIGURE 10:**
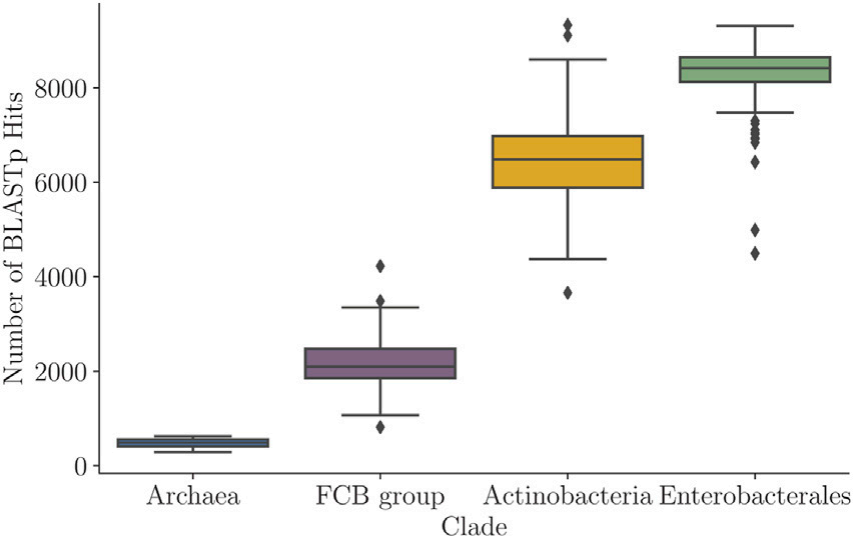
Distribution of numbers of the BLASTp hits per query in the four clades (Table 1) shown as box plots of the numbers of the BLASTp hits per query.

Besides the variability in the Kimura distance distributions, the four prokaryotic clades also showed clade-specific variability among query genes with respect to the numbers of homologs detected in similarity searches. A distribution of the number of BLASTp hits (prior to any filtering) in each of the four clades is shown in Figure 10, while the percentages of query genes (per genome) that had at least N BLASTp hits where N varies from 0 to 5,000 hits are shown in Supplementary Figure S13.

Naturally, the number of hits per query was largely proportional to the number of genomes within a clade (Table 1; Figure 10). On the other hand, the cumulative distributions (Supplementary Figure S13) increased very quickly and plateau early on, first for *Archaea* (1,125 genomes) and then the *FCB* group (3,306 genomes). In *Enterobacterales* (6,311 genomes) and *Actinobacteria* (8,097), the cumulative distributions grew much more slowly. Still, *Actinobacteria*’s distribution (which has *more* genomes) grew significantly faster than that of *Enterobacterales*. For example, the likelihood that a query in *Enterobacterales* got *at least* 1,000 BLAST hits was ≈83% , compared to only 60% in *Actinobacteria*.

### 4.6 Visualization of the StartLink Data Analysis

**FIGURE 11:**
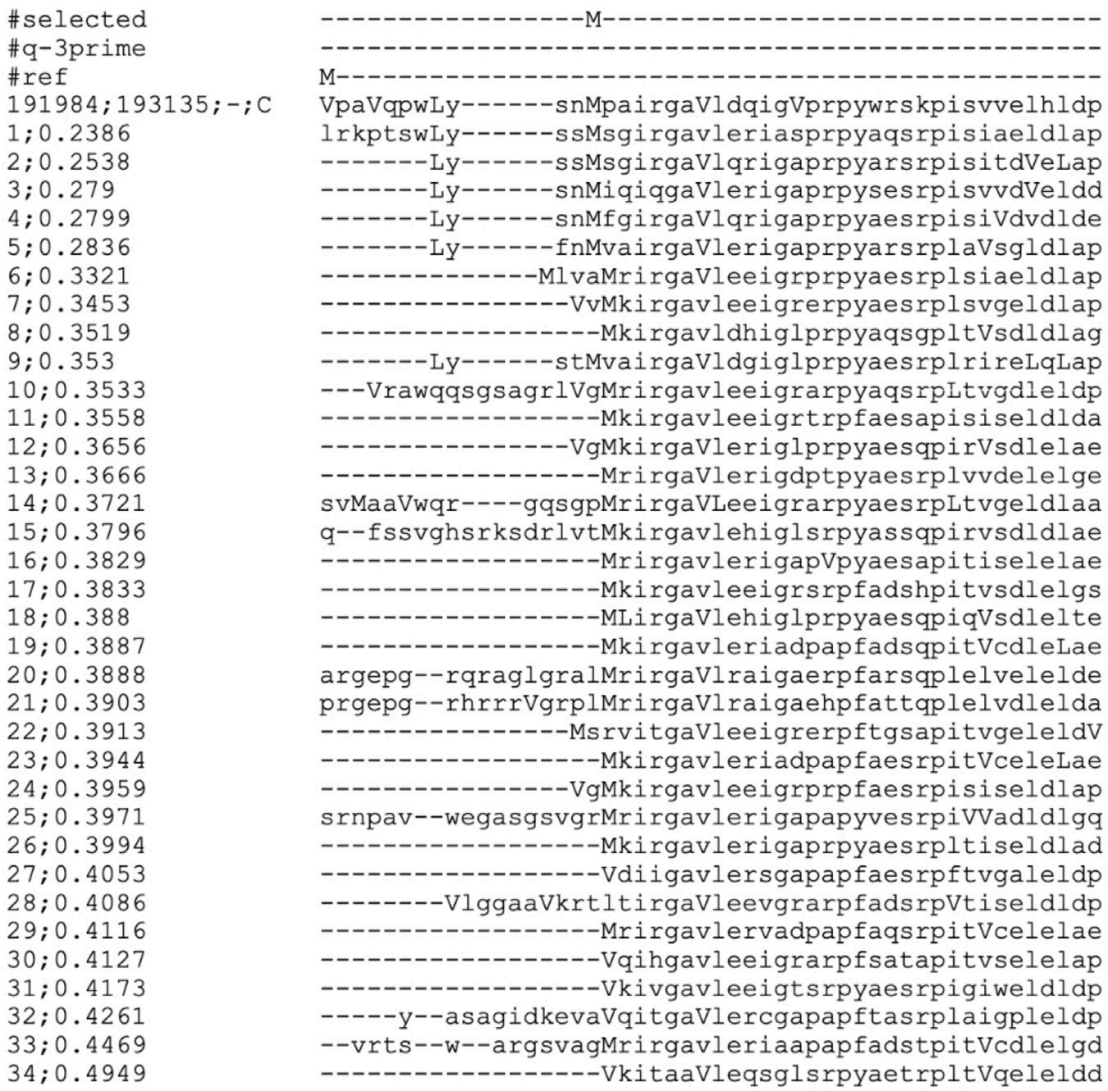
MSA for the gene adhE1 Rv0162c in *M. tuberculosis*, the Actinobacteria clade.

The multiple sequence alignments used for the StartLink inference could be of interest for visual inspection of the pattern of conservation. For example, an MSA made for a gene adhE1 Rv0162c in *M. tuberculosis* showed a case where StartLink+ prediction was different from the annotated gene start (Figure 11).

The top row amino acid sequence (line 4) is the translated query sequence, followed by the sequences of selected homologs. Capital M, V, and L letters represent methionine, valine, and leucine coded by ATG, GTG, and TTG, respectively. Lowercase v and l represent valine and leucine coded by non-GTG or non-TTG codons, respectively.

Annotated start of this gene (“#ref”) was the GTG-coded valine, while StartLink+ was predicted as start the downstream methionine (“#selected”). We see that the prediction made by StartLink+ had a high conservation of both the gene start and the immediate downstream region. Conversely, the annotated start was positioned in a highly non-conserved upstream region (more MSA examples are shown in Supplementary Note 7).

## 5 Discussion

### 5.1 Comparison of Gene Starts Predicted by Different Tools

We used several existing sets of genes with experimentally verified starts (Table 1) for benchmarking of error rates in gene start prediction (Table 2). We saw that StartLink+ was the most accurate tool for the genes where predictions were made. Therefore, we used StartLink+ for analysis of larger genomic sets where comparisons would indicate room for improvement of the individual tools (Figure 7).

The genomic percentage of genes with differences in predicted starts turned out to depend on genome GC content. This dependence appeared to have the same pattern when we compared either PGAP or Prodigal to StartLink+ (Figure 7). Large differences with Prodigal were observed also for *Actinobacteria*, *Archaea*, and *FCB* groups. Still, the difference between StartLink+ and Prodigal for *Actinobacteria* had a peak at 67% GC (Figure 7). Note that the plots of the difference averaged among all the genomic sets (the dashed lines) were computed by using all the genomes rather than using just the data from the colored graphs with equal weights. The average genomic percentage of genes with gene-start differences between StartLink+ and RefSeq annotation was determined for the set of 5,488 representative genomes. This percentage also increased with an increase in the genome GC content (Figure 1).

The frequency of making gene start prediction errors in genomes with high GC could be elevated due to the longer average LORFs. This factor should have a stronger influence on *ab initio* gene finders. Another noise component is acting on alignment-based methods. It could be related to variations in distributions of database orthologs across Kimura distances (Supplementary Figure S12). We showed that StartLink+ performed reliably across the range of Kimura distances (Supplementary Note 2). Particularly, to account for the gene-specific speed of gene sequence evolution, the selection of targets was gene-specific rather than being genome-specific. This approach implemented in StartLink could lead to differences in the sets of target sequences used in PGAP. Still, this factor was unlikely to make a concerted impact on the frequency of differences that would depend on genome GC content.

In comparison of PGAP and StartLink+, we also considered the frequency of differences in the groups of genes whose starts were predicted at algorithmic steps A, B, and C (Supplementary Figure S10). Lower frequencies of differences were observed at step A as could be expected. In the genes of group A in a given LORF, we had a single start candidate upstream to a conserved region predicted to be protein-coding.

### 5.2 StartLink and StartLink+ Do Not Make Start Predictions for Some Genes

**FIGURE 12:**
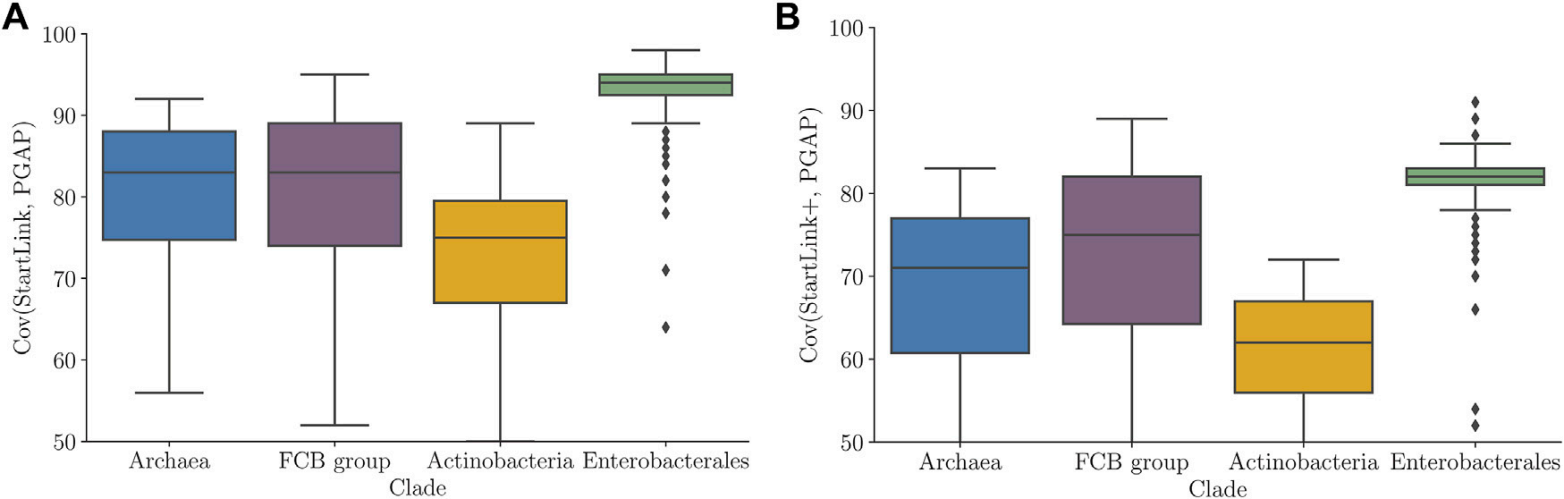
Coverage rates of **(A)** StartLink and **(B)** StartLink+ for the four clades. The analysis was done for the same sequence data as in Figure 7.

The StartLink’s overall genomic coverage was 85% on average (Figure 12A). The *Enterobacterales* average, 92% per genome, was, however, significantly higher than 80–83% average observed for the remaining three clades. The coverage per genome should depend on a phylogenetic position of the species as well as the pattern of selection of the evolutionarily close or distant species for whole-genome sequencing. The percentage of genes produced a certain number of significant BLASTp hits in similarity search with their protein translation as queries provide an upper bound for the genomic coverage. The genomic percentage of queries that had at most n BLASTp hits, n∈[0, 40], is shown in Supplementary Figure S14. We saw that on average, 10% of genes in *Archaea*, 12% of genes in *Actinobacteria*, and 12% of genes in the *FCB* group genomes had fewer than 10 BLASTp hits, while only 3% of *Enterobacterales* genes had fewer than 10 hits. These hits, however, might not land within the desired Kimura distance intervals to the nucleotide query and to each other. We see that a large part of the loss of coverage in each of the clades could be traced back to the low number of the BLASTp hits.

We saw that StartLink+ predicted gene starts for 73% genes (on average) in a genome (Figure 12B). The differences between clades again reflect the difference in abundance of sequenced genome, for example, between *Enterobacterales* and *Archaea*. However, the ∼12% drop on average between StartLink and StartLink+ corresponds to “mutual filtering” of false positive by StartLink and GeneMarkS-2. The largest change across the clades was in *Actinobacteria*’s coverage dropped by 16%.

### 5.3 Experiments With Different Types of Integration of Gene Finding and Gene Start Finding Tools

To investigate how the accuracy of gene start prediction depends on the choice of gene finding tools for integration, we have experimented with the integration of the following pairs: StartLink and Prodigal, and GeneMarkS-2 and Prodigal. The accuracy of the use of these integrated pairs was compared with StartLink+ integrating StartLink and GeneMarkS-2. The integrated predictions made by each pair of tools were recorded only when predictions of both tools matched each other. The results showed that the pairs integrated as “independent streams” of information, multiple-alignment-based, and *ab initio*-based delivered more accurate predictions (Table 2). It was demonstrated that StartLink+ had the best accuracy among the integrated pairs of tools.

### 5.4 Effects of Restrictions on the Number of Targets per Gene

To reduce the running time of StartLink, we limited the maximum number of allowed targets used in MSA (currently, N=50 ). We could select at most N target sequences and continue with further selection within the MSA (Supplementary Note 6). The average number of targets per query after a full StartLink run could differ significantly between clades (Figure 3), especially when comparing *Archaea* and *Enterobacterales*.

A possible reason for *Enterobacterales* genes to have a high average number of selected targets was the larger spread of the Kimura distances than other clades (Supplementary Figure S12). The average number of targets within *Archaea* was frequently reaching as low as 10 targets per query. This was partly due to a small number of sequenced genomes in this clade, making it less likely that we find enough sequences within the right Kimura range.

We should note that for the set of genes with verified starts, the observed differences in the number of targets per query did not translate into a difference in the StartLink accuracy. For example, when StartLink was run with N=50 , both *Archaea* (*H. salinarum* and *N. pharaonis*) ended up with 20 targets per query on average, compared to the *E. coli* 40 targets per query. However, for *H. salinarum* and *N. pharaonis*, we observed gene-start errors in 3 and 2% of genes, respectively, while for *E. coli*, it was in 5% of genes.

To assess the StartLink performance on *Archaea* with a low average number of target per query, we decreased N to 20. This change produced 10 to 15 targets per query for both *Archaea* species. As a result, we saw a slight increase in the percentage of erroneous predictions for *H. salinarum* (by 0.6%) and a decrease for *N. pharaonis* by 0.7%. For all the sets of genes with verified starts, we saw 0.5% change (on average per genome) when N was changed. This outcome demonstrated that StartLink was robust with respect to changes of N .

## 6 Summary

Existing computational gene finders differ in gene start predictions in 15–25% of genes in a prokaryotic genome while making accurate predictions of protein-coding open-reading frames (unambiguously defined by stop codons). Our task was to improve gene start prediction. First, we developed StartLink that infers gene starts from patterns of evolutionary conservation derived from alignments of homologous genomic and protein sequences. Next, we introduced StartLink+ that combined predictions made independently by StartLink and an *ab initio* gene finder GeneMarkS-2. We have shown that StartLink+ delivered low error rates in gene start predictions (∼1%) for a sufficiently high percentage of genes in a genome (∼73% on average). StartLink and StartLink+ could be used i) in studies on improving prokaryotic genome annotations, ii) for more accurate inference of sequence patterns around gene starts, and iii) in studies of regulatory sequences selected in evolution near gene starts to control diverse gene expression mechanisms.

## Data Availability

The original contributions presented in the study are included in the article/Supplementary Material, and further inquiries can be directed to the corresponding authors.
